# Factors associated with prelacteal feeding in North Eastern Ethiopia: A community based cross-sectional study

**DOI:** 10.1186/s13006-016-0073-x

**Published:** 2016-05-17

**Authors:** Nigus Bililign, Henok Kumsa, Mussie Mulugeta, Yetnayet Sisay

**Affiliations:** Department of Midwifery, Faculty of Health Sciences, Woldia University, Woldia, Ethiopia; Department of Health Education and Behavioral Sciences, Institute of Public health, University of Gondar, Gondar, Ethiopia

**Keywords:** Prelacteal, Factors, Ethiopia

## Abstract

**Background:**

In spite of the negative impact of prelacteal feeding on the growth and development of children, it is widely practiced in Ethiopia. This study aimed to assess prelacteal feeding practices and associated factors among mothers of children aged less than 24 months in the North Wello zone.

**Methods:**

A quantitative community based cross-sectional study was employed during March 2015. Eight hundred and forty four (844) mother-child pairs were selected by multi-stage sampling technique. Data were collected by face-to-face interview. Descriptive statistics, binary and multiple logistic regression analyses were employed to identify factors associated with prelacteal feeding practice. Variables with a *p*-value <0.05 were identified as statistically significant factors.

**Results:**

The prevalence of prelacteal feeding was 11.1 % (95 % confidence interval [CI]: 9.0, 13.0). Colostrum discarding (adjusted odds ratio [AOR]: 8.7; 95 % CI (3.8, 20.1)) and lack of counseling about breastfeeding (AOR: 2.6; 95 % CI 1.27, 5.4) were the factors associated with prelacteal feeding. The major reasons stated for providing prelacteal feeds were “culture” and “do not have enough milk”.

**Conclusion:**

Prelacteal feeds are offered to nearly one child in every ten in the North Wello zone. Colostrum removal and lack of counseling on breastfeeding at antenatal care visit are important positive predictors of prelacteal feeding practice. Awareness of the risks associated with prelacteal feeding, promotion of counseling on breastfeeding and the health benefit of colostrum during antenatal care visits are recommended interventions to reduce prelacteal feeding practices in the study areas.

## Background

Breastfeeding is the natural way of providing young infants with the nutrients they need for healthy growth and development [1]. Infants should be exclusively breastfed for the first six months of life to achieve optimal growth, development and health [2]. Exclusive breastfeeding means that the infant receives only breast milk without any additional food or drink, not even water. Exclusive breastfeeding for the first six months of life has several advantages including a lower risk of gastrointestinal infection for the baby, more maternal weight loss after birth and delayed return of menstrual periods [3].

The World Health Organization (WHO) and United Nations Children’s Fund (UNICEF) recommend initiating breastfeeding within one hour of birth and exclusively breastfeeding for the first six months. It is also recommended that breastfeeding should be continued for two years or more together with safe, nutritionally adequate, age appropriate, responsive complementary feeding starting from six months [4].

A prelacteal feed is any food except mothers’ milk provided to a newborn before breastfeeding is established. Practice of prelacteal feeding is a major barrier to exclusive breastfeeding [5]. Optimal breastfeeding of infants under two years of age has a significant impact on child survival, with the potential to prevent over 800,000 deaths in children under five (13 % of all deaths) in the developing world [6].

A cross-sectional study in western Nepal showed that 30.6 % of mothers reported giving prelacteal feeds to their infants. The most popular prelacteal foods were formula milk (41.7 %), cow or buffalo milk (26.6 %) and sugar/glucose water (12.4 %) [7].

In Ethiopia, currently 24 % of infant deaths are due to poor breastfeeding practices [8]. The 2011 Ethiopian Demographic and Health Survey (EDHS) showed that 27 % of children were given prelacteal foods within the first three days of postpartum period. It is more commonly practiced by Ethiopian rural residents (27.5 %) than urban residents (24.2 %). Somali (72.5 %) and Amhara (47.8 %) regional states had the highest prevalence of prelacteal feeding practice [9].

A community-based study conducted in East Ethiopia showed that the prevalence of non-exclusive breastfeeding was 28.3 % [10]. A cross-sectional study conducted in Jimma showed that more than three quarters of mothers practice sub-optimal breastfeeding. Furthermore 37 % of mothers initiated breastfeeding greater than one hour after delivery, which was significantly associated with not attending formal education [11]. Another community-based cross-sectional study conducted in Bahir dar city showed that giving birth at health facilities and receiving infant feeding counseling were predictors of exclusive breastfeeding practice [12].

Prelacteal feeding is associated with increased neonatal illnesses and mortality [13]. Colostrum is the first breast milk secreted in the first three days of postpartum period and contains immunoglobulin and other biological components that provide natural immunity against many bacteria and viruses [14]. Prelacteal foods can make the newborn susceptible to infection by interfering with breast milk production. Furthermore, contaminated feeds and utensils used for the introduction of prelacteal foods can cause infection of the newborn especially due to the permeability of the immature neonatal gut lining [15]. In addition, mother-baby bonding may be interrupted by prelacteal feeding as it decreases skin-to-skin contact [16].

A study conducted in Raya Kobo district of Ethiopia showed that the most common prelacteal feeds were sugar solution and raw butter. The reasons stated to give prelacteal feeds were to clean the infant’s stomach and to prevent ‘evil eye’ and illness [17]. Despite the advantage of early initiation in breastfeeding for the growth and development of newborn babies, prelacteal feeding remains widely practiced in Ethiopia. Little is known about the predictive factors for prelacteal feeding practices and therefore studying these may ultimately help to discourage introduction of those feeds for newborns. The findings of this study will help policy makers and program designers to produce appropriate educational material and interventions to optimise infant feeding practices. The aim of this study therefore was to assess prelacteal feeding practice and associated factors in Woldia, Kobo and Lalibela towns of the Amhara region in Ethiopia.

## Methods

### Study setting, design and participants

This study was conducted in the North Wollo zone (Woldia, Kobo and Lalibela towns) of North Eastern Ethiopia during March 2015. North Wollo zone is one of the ten zones of the Amhara region, located 520 km north-east of Addis Ababa. Based on the 2007 census conducted by the Central Statistical Agency (CSA) of Ethiopia, this zone has a total population of 1,500,303 of whom 752,895 were men and 747,408 were women. There are 64 functional health centres and three hospitals in North Wollo zone. Woldia, Kobo and Lalibela towns have sixteen, five and four health extension workers respectively [*North Wollo zone Health Department report, 2015: unpublished*]. A quantitative community based cross-sectional study was employed. All mothers who had children less than 24 months of age were the source population of this study.

### Sample size determination

The sample size was determined using a formula for the estimation of a single population proportion as follows [18]:

$$ n=D\left[\ \frac{{\left(Z\frac{\alpha }{2}\right)}^2\ P\left(1-P\right)}{d^2}\ \right] $$

Where *n* = required sample size, Z = critical value for normal distribution at 95 % confidence level (1.96), *P* = 50 %, d = 0.05 (5 % margin of error), D = 2 (design effect), and with an estimated non-response rate of 10 % produced a final sample size of 844.

### Sampling procedure

Multi-stage sampling was employed to select the 844 study subjects. A pre-survey was conducted before the actual day of data collection to determine which households had the target mother-child pairs. 6,013 households contained the targeted mother-child pairs in the selected eight kebeles (the smallest administrative unit in Ethiopia). Kebeles of the towns were selected by simple random sampling (SRS). At the kebele level households were selected by systematic sampling method. According to the population proportion in the study areas; 420, 224 and 200 mothers were selected from Woldia, Kobo and Lalibela towns respectively. A starting point was identified based on the help of health extension workers.

### Inclusion and exclusion criteria

From each household unit one eligible mother who had a biological child aged less than 24 months was selected. Non-biological mothers and mothers who were unable to communicate due to disability or any other health problem were excluded from the study.

### Data collection procedure

Data were collected using a pre-tested, structured, interviewer-administered questionnaire adapted from the Ethiopian National Nutrition Survey questionnaire [16]. The adapted questionnaire was modified according to the research objective and the actual setup. The questionnaire was prepared first in English, translated into Amharic, and then back into English by fluent speakers of both languages to check its consistency. The data was collected by six diploma midwives and three Bachelor of Science degree holder midwives were recruited as supervisors. The data collectors and the supervisors were trained for three days (including practical work) by the principal investigator (Nigus Bililign).

### Study variables

#### Dependent variable

Prelacteal feeding practices among mothers of children aged less than 24 months. Prelacteal feeding was defined as providing foods and/or drink other than human milk for the infant before breastfeeding was established [5].

#### Independent variables

The adapted questionnaire had three sections (both closed and open-ended questions). These were: socio-demographic characteristics (age, marital status, educational status, religion), maternal factors (antenatal care visit, parity, place of delivery) and child feeding practices (prelacteal feeding, colostrum feeding). The variable ‘Antenatal Care Visit’ was defined as receiving at least one visit of health facility during the associated pregnancy. The ‘Postnatal Care Visit’ variable was defined as receiving at least one visit within the six-week postpartum period. ‘Untrained traditional birth attendant’ was used to describe individuals who provided delivery services without having formal training in basic mother and child health care.

### Data processing and statistical analysis

The data was checked for completeness and inconsistencies. It was also cleaned, coded and entered to the SPSS version 20.0 computer program. Univariate binary logistic regression analysis was performed to assess the association between each single independent variable and the dependent variable (prelacteal feeding yes/no). Multivariable logistic regression was performed to control the possible confounding factors. Variables with a *p*-value < 0.25 in the binary logistic regression analysis were used in the multivariable logistic analysis, with the same binary dependent variable (prelacteal feeding yes/no). The Hosmer-Lemeshow goodness-of-fit with enter procedure was used to test for model fitness. Adjusted Odds Ratios (AOR) with a 95 % confidence interval were estimated to assess the strength of associations and statistical significance was declared at a p-value <0.05.

### Ethical considerations

The study was approved by the Institutional Research Review Board of Woldia University. An official letter was written from Woldia University to the Woldia, Kobo and Lalibela town Administration Offices. Then a permission and support letter was written to each selected Kebele. Informed verbal consent was received from the participants before the interview. The participants were also assured about the confidentiality of the information they provided.

## Results

### Socio-demographic characteristics of the study participants

A total of 782 mother-child pairs were included in the study, yielding a response rate of 92.6 %. The mean age of respondents was 27.02 (± SD 5.47) and ranged from 15 to 48 years. Ethiopian Orthodox Christianity was the dominant religion accounting for 84.7 %. The vast majority (96.5 %) of respondents were ethnically Amhara. About 73 % of the respondents attended formal education and 60.5 % of mothers were housewives (Table 1).

**Table 1:** Socio-demographic characteristics of mothers with children aged 24 months or less in the Woldia, Kobo and Lalibela towns of North- Eastern Ethiopia, March 2015

Variables	Frequency (n)	Percent (%)
Age of mother (*n* = 782)		
<20	88	11.3
20–34	598	76.5
>34	96	12.3
Mothers’ educational status (*n* = 782)		
No formal education	212	27.1
Formal education	570	72.9
Mothers’ marital status (*n* = 782)		
Single	31	4.0
Married	681	87.1
Divorced	66	8.4
Widowed	4	0.5
Religion of mother (*n* = 782)		
Orthodox	662	84.7
Muslim	109	13.9
Protestant	10	1.3
Others	1	0.1
Mothers’ ethnicity (*n* = 782)		
Amhara	755	96.5
Tigray	23	2.9
Others	4	0.5
Mothers’ occupation (*n* = 782)		
House wife	473	60.5
Student	43	5.5
Merchant	112	14.3
Government Employee	101	12.9
Others	52	6.6
Sex of last child (*n* = 782)		
Male	413	52.8
Female	369	47.2

### Maternal and child health service uses

Ninety-four percent of respondents had attended at least one antenatal care visit, 10.5 % gave birth at home and more than a half (53.7 %) attended at least one postnatal care visit after the birth of their last child. More than half (52.4 %) of the mothers did not receive counseling on breastfeeding during their antenatal care visit(s). Regarding mode of delivery, the vast majority (83.8 %) of the respondents gave birth vaginally. During delivery, 11.4 % of the mothers were assisted by untrained traditional birth attendants (Table 2).

**Table 2:** Maternal and child health service utilization among mothers having children aged 24 months or less in the Woldia, Kobo and Lalibela towns of North- Eastern Ethiopia, March 2015

Variables	Frequency (n)	Percent (%)
Antenatal care visit (*n* = 782)^a^		
Yes	735	94.0
No	47	6.0
Counseling on breastfeeding during antenatal care visit (*n* = 782)		
Yes	325	41.6
No	410	52.4
Place of delivery (*n* = 782)		
Health institution	700	89.5
Home	82	10.5
Mode of delivery (*n* = 782)		
Vaginal	655	83.8
C/S	127	16.2
Assistant during delivery (*n* = 782)		
Health professional	693	88.6
Others	89	11.4
Postnatal visit (*n* = 782)^a^		
Yes	420	53.7
No	362	46.3

^a^at least one visit

### Child feeding practices

Almost all respondents (98.3 %) had ‘ever’ breastfeed their last children. Of those who had ever breastfeed, 601 (76.9 %) mothers initiated breastfeeding within one hour of birth.

Of the total mothers, 87 (11.1 %, 95 CI: 9.0 %, 13.0 %) gave prelacteal foods to their children. The most common prelacteal foods were sugar solution (60 %) and Ersho (18 %). Raw butter, plain water and cow milk constitutes 13 %, 5 % and 4 % respectively. Culture (54 %) and not having enough milk (40.2 %) were the main reasons for prelacteal feeding practice reported by the respondents. About 78 % of mothers were influenced by other individuals to practice prelacteal feeding. The influential individuals were grandmothers of the index children (57.3 %), untrained traditional birth attendants (38.2 %) and husbands of the respondent (4.4 %). Of the total respondents, 87 (11.1 %) mothers discarded their first milk (colostrum) (Table 3).

**Table 3:** Feeding practices among mothers of children aged 24 months or less in the Woldia, Kobo and Lalibela towns of North- Eastern Ethiopia, March 2015

Variables	Frequency (n)	Percent (%)
Ever breastfeeding (*n* = 782)		
Yes	769	98.3
No	13	1.7
Early initiation of breastfeeding (*n* = 782)		
Yes	601	76.9
No	168	21.5
Have you ever heard about breastfeeding? (*n* = 782)		
Yes	504	64.5
No	278	35.5
Where did you get the information? (*n* = 504)^a^		
Health facility	429	85.1
Family	76	15.0
Mass media	73	14.5
Others	32	6.3
Type of information (*n* = 504)^a^		
Benefits of breastfeeding	366	72.6
Techniques of breastfeeding	312	61.9
Breast milk initiation time	232	46.0
Others	85	16.9
Prelacteal feeding (*n* = 782)		
Yes	87	11.1
No	695	88.9
Reasons for prelacteal feeding (*n* = 87)^a^		
Culture	47	54.0
No enough milk	35	40.2
Breast pain	2	2.3
I was sick	5	5.7
Others	3	3.4
Influenced to give prelacteal feeding (*n* = 87)		
Yes	68	78.1
No	19	21.83
Influential individual for prelacteal feeding (*n* = 68)		
Husband	3	4.4
Traditional birth attendant	26	38.2
Child’s grandmother	39	57.3
Colostrum feeding (*n* = 782)		
Yes	695	88.9
No	87	11.1
Reasons for not feeding colostrum (*n* = 87)		
Not clean	23	26.4
Difficult for the baby	13	14.9
Will cause abdominal pain	32	36.8
Not important	6	3.4
Others	3	6.9

^**a**^variables which have multiple responses

### Factors associated with prelacteal feeding

In the univariate logistic regression analysis early initiation of breast feeding, colostrum feeding, counseling about breastfeeding during antenatal care visit, home delivery and the presence of untrained assistant during delivery were associated with prelacteal feeding practice.

In multivariable logistic regression analysis only lack of colostrum feeding and lack of breastfeeding counseling at antenatal care visit remained predictors of prelacteal feeding. Mothers who did not give colostrum to their babies were almost nine times more likely to practice prelacteal feeding as compared with mothers who gave colostrum (AOR:8.7; 95 % CI 3.8, 20.1). Mothers who were not counseled about breastfeeding at antenatal care were 2.6 times more likely to practice prelacteal feeding as compared with their counterparts (AOR: 2.6; 95 % CI 1.27, 5.4) (Table 4).

**Table 4:** Factors associated with prelacteal feeding practices among mothers of children aged 24 months or less in the Woldia, Kobo and Lalibela towns of North- Eastern Ethiopia, March 2015

Variables	Prelacteal feeding n (%)	Crude odds ratio (95 % CI)	Adjusted odds ratio (95 % CI)
Age of mother			
<20	14 (15.9)	1	1
20–34	63 (10.5)	0.6 (0.30, 1.16)	1.95 (0.41, 9.22)
>34	10 (10.4)	0.6 (0.26, 1.46)	2.9 (0.47, 17.91)
Mothers’ educational status			
No formal education	29 (13.7)	1.39 (0.86, 2.25)	1.1 (0.49, 2.53)
Formal education	58 (10.2)	1	1
Early initiation of breast milk			
Yes	49 (8.2)	1	1
No	34 (20.2)	2.8 (1.70, 4.61)	1.9 (0.89, 4.02)
Colostrum feeding			
Yes	54 (7.8)	1	1
No	33 (37.9)	7.3 (4.30, 12.12)	8.7 (3.82, 20.10)^a^
Counseling on breastfeeding at antenatal care visit			
Yes	22 (6.8)	1	1
No	49 (12)	1.9(1.12, 3.16)	2.6 (1.27, 5.42)^a^
Sex of index child			
Female	35 (9.5)	1	1
Male	52 (12.6)	1.4 (0.93, 2.15)	1.1 (0.52, 2.20)
Place of delivery			
Health facility	55 (7.9)	1	1
Home	32 (39)	7.5 (4.45, 12.65)	3.4 (0.63, 19.51)
Assistant during delivery			
Others^b^	32 (36)	6.5 (3.89, 10.87)	1.3 (0.24, 7.12)
Health professional	55 (8)	1	1

^a^Statistically significant variables at p-value = 0.05, *CI* confidence interval, ^b^ TBAs and family members, Hosmer- Lemeshow goodness-of-fit = 0.389

## Discussion

In spite of the negative impact of prelacteal feeding on the growth and development of children, it remains widely practiced in Ethiopia [9]. We found that the prevalence of prelacteal feeding in Woldia, Kobo and Lalibela towns was about 11 %. Colostrum disposal and lack of breastfeeding counseling at an antenatal care visits were predictors of prelacteal feeding practice.

Breastfeeding is a universal practice in the study area, but it has been sub-optimal due to the introduction of prelacteal foods. This study revealed that the prevalence of prelacteal feeding in Woldia, Kobo and Lalibela towns was 11.1 %, lower than a study conducted in the Raya kobo district (38.8 %) [18]. This difference might be due to the fact that in the case of the Raya kobo district 86 % of the study subjects were from rural areas, whereas in this study participants were from the urban part of North Wello Zone. Hence mothers who reside in the towns have better access to maternal and child health services. Similarly prevalence of prelacteal feeding in this study was lower compared to a report of the Amhara regional state (11.1 % versus 47.8 % respectively). This difference can be explained by the fact that our study includes only one zone of the Amhara region whereas the report is from all areas of the region. A similar prevalence of prelacteal feeding (10.4 %) was reported from the southern region of Ethiopia. The highest prevalence of prelacteal feeding practices in Ethiopia was reported from the Somali regional state of Ethiopia (72.5 %) [9].

This study showed that mothers who discard colostrum were almost nine times more likely to practice prelacteal feeding compared to mothers who gave colostrum to their children. A qualitative study conducted in the Raya kobo district showed that colostrum is thought to cause abdominal cramps and raw butter is thought to clean infants’ stomachs. Therefore, untrained traditional birth attendants advised mothers to discard colostrum and feed their infants with raw butter before breastfeeding initiation [18].

In the present study, breastfeeding counseling during an antenatal care visit was associated with a reduced likelihood of prelacteal feeding. A cross-sectional study in Maharashra, India showed that mothers who did not receive counseling on breastfeeding at antenatal care visits gave more prelacteal feeds compared to mothers who did receive counseling [19]. Similar findings were reported from Kolkata, India [20]. A study conducted in the Harari region health facilities revealed that mothers who did not receive antenatal care services were 2.6 times more likely to practice prelacteal feeding than their counterparts [21]. A study conducted in the city of Bahir Dar showed that institutional delivery and receiving infant feeding counseling were predictors of exclusive breastfeeding [12].

In this study, birth at home was not significantly associated with practice of prelacteal feeding. This is consistent with findings from Nepal [5]. However, a different study conducted in Kolkata found that home delivery was significantly associated with the practice of prelacteal feeding [20]. When birth occurs at home local community members may advise the use of prelacteal feed, thus affecting newborn feeding practices. In this study, 78.1 % of mothers were influenced by other individuals to introduce prelacteal foods to their children. The influential individuals were children’s grandmothers, traditional birth attendants and husbands. This implies that home delivery and influential individuals may be associated with an increased practice of prelacteal feeding.

A strength of this study was that it employed a community-based approach to minimize selection bias. However, a limitation of this study was that, information obtained from mothers is subject to recall bias. Additionally, we cannot indicate the direction of causation to the associative relationship because of the study’s cross-sectional study design.

## Conclusion

Prelacteal feeding is practiced in Woldia, Kobo and Lalibela towns, but is not prevalent as compared to the rural settings in the Amhara region. In this study, nearly one in ten newborns had received prelacteal foods. Colostrum disposal and lack of counseling about breastfeeding at antenatal care visits were factors significantly associated with prelacteal feeding practices. Findings of this study can be used to promote optimal breastfeeding practices. Optimal breastfeeding can assure growth, development and health of young children. Awareness of the factors associated with prelacteal feeding and the promotion of health benefits associated with colostrum and early breastfeeding during antenatal care visits are recommended interventions to reduce prelacteal feeding practices in the Woldia, Kobo and Lalibela towns. Interventions should also target health professionals within the study area. Further qualitative research is needed to determine how best to utilise the findings of this study. Gaining more details about specific prelacteal practices would enable more appropriate educational messages targeted at specific behaviors and beliefs.

## Abbreviations

AOR: adjusted odds ratio
CI: confidence interval
COR: crude odds ratio
CSA: central statistical agency
EDHS: Ethiopian demographic and health survey
Ersho: a traditional baking soda prepared by incubating the flour and double distilled water
IYCF: infant and young feeding practice
MDG: millennium development goal
SRS: simple random sampling
UNICEF: united Nations children’s fund
UTBA: untrained traditional birth attendant
